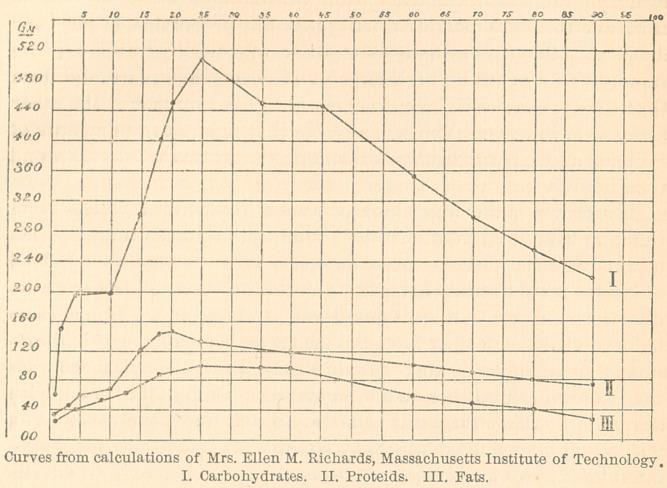# Foods

**Published:** 1894-03

**Authors:** R. W. Greenleaf


### FOODS.[1]

BY PROFESSOR R. W. GREENLEAF.

Mr. President and Gentlemen,—I ask your indulgence in presenting these few remarks this evening; they are not to be taken as a paper, but simply as a substitute for one. A few days ago, I was asked to say something at this meeting in place of one of your members. I asked my friend what I should bring before

---

[1] A Smoke-Talk before the Harvard Odontological Society, September 28, 1893.

you and whether you would be likely to be interested in the subject of "Foods." He said, "By all means."

I shall assume that much of the knowledge of foods is thoroughly familiar to you,—that is, the parts relating to the first features of digestion,—I mean the importance of having and using good organs of mastication, but I should like to bring before you certain other phases of this question which the profession of dentistry would do well to review from time to time and to which they have an especial opportunity of calling the attention of the laity. There are certain food questions which are of importance to whole communities, such as towns and cities, on which professional men should be thoroughly equipped, and it seems to be wise to bring these matters before you.

One of these questions might be called the municipal phase of the food question. I do not think most of us realize the importance of securing an absolutely pure food-supply, or of the difficulty of obtaining one. It is absolutely necessary for us to have good food. Certain of the foods may be brought to us in good condition, for instance the various starches of the cereals and dry foods. Grains, flours, etc., may be kept for an indefinite time without undergoing any deleterious change, but it is not easy to keep such foods as meats, milk, and vegetables. And we have also another feature to guard against in this matter of food-supply,—that is, adulteration. Certain of these dry foods may be adulterated by various things, and we also know that milk and substances of that nature, besides being adulterable, may undergo such changes as the simple putrefactive change. It is to prevent these deleterious changes that arise in these products that laws are made and our State boards of health have the power to enforce them. They have inspectors who from time to time visit markets and places where foods are offered for sale, and if any adulterations are found, those who sell them are punished according to law. In this connection it is curious to note the contrivances that are adopted by dishonest men for the purpose of adulteration. Take, for instance, coffee, from which we have been deriving so much pleasure this evening. Sometimes pease are steamed and browned over and made to resemble the coffee beans. When ground one would hardly detect this imposition with the eye. Again, certain of these men adulterate coffee with brown bread, which is crushed and tightly pressed into moulds of the size and shape of the coffee bean, so that it is difficult to distinguish it from the genuine article. Of course, microscopic and other tests enable one to distinguish them easily.

Besides the State boards of health, we have local boards who adopt similar precautions and appoint inspectors with authority to prosecute those who are found offering for sale adulterated foods, and we hear from time to time of the vast amount of work which our own inspector is doing. We also have inspection of other departments, as, for instance, the slaughter-houses and certain manufactories, etc., but with all this we are sadly deficient as regards proper care for these supplies. The national government has an especial corps of officers for the inspection of animals to be sent to foreign ports, and the system of inspection is so thorough that it is difficult to find diseased meat of any description leaving this country, but it is not difficult to find tainted meats in our markets. We are not as well protected in this matter of meats and foods by our own government as we are protecting the people of foreign countries.

It is incumbent upon us to see that our meat-supply is kept in good condition, and that we should use our influence to see that our markets be daily inspected by competent people, so that under no circumstances should meat be sold which is liable to produce disease. We can assist in getting a better food-supply by spreading knowledge in regard to things of this character, and when people understand these things more fully they will adopt stronger measures against those who sell tainted canned foods and meats and adulterated milk, butter, etc. We often hear of cases of sudden poisoning following dinners, repasts, and the like,—I do not anticipate anything of that kind after this delightful dinner,—but we know that certain of the foods used on such occasions may have contained some poisonous substance. Cases of poisoning may arise from the use of canned goods in which have been introduced substances for the preservation or coloring of the foods. A more common source of cases of sudden poisoning from eating canned or tainted meats, fishes, etc., is the presence of an active alkaloid poison, comparable to the alkaloids of such drugs as digitalis, conium, veratrum, etc., which may develop in decomposing organic matter as a result of the growth of bacteria. These poisons are called ptomaines, and are extremely active agents. Therefore, we should see that everything is done to secure a most perfect supervision of our markets, canning establishments, etc., and that no foods are sold which will in any way endanger the public health.

Professor Sedgwick has shown that milk in its early stages, directly after being obtained from a healthy cow, is free from any kind of germ-life; also, that soon afterwards it becomes contaminated with germs which get into it from the surrounding air and multiply with extraordinary rapidity, so that in a short time the milk may be completely spoiled. These germs may be harmless, but just in the same way that harmless germs are taken in so are the poisonous ones likely to get in, supposing they are about, so that it is necessary for us to take the greatest care in such particulars, and not simply take it ourselves, but to help spread a general knowledge throughout the community of such matters. The ignorance of people in intelligent communities on this subject is surprising. Milkmen, even in a city like Boston, have been found to be cooling their milk in cellars that were improperly drained, and in many houses you will find that such things as cakes and puddings are allowed to be cooled in damp and improperly-aired places.

Now, I would like to say a few words in relation to quite another department of the food question which it seems to me is not generally comprehended, and which might be understood with considerable advantage. You are familiar with the fact that food, as such, consists of elementary substances of four different kinds. The first of these is called the carbohydrates, and under this head are included such foods as the starches and sugars found abundantly in cereals; also to some extent in animal foods. Next in the series are the proteids, which are represented by the albuminoids that are contained in the various meats, but are also found in vegetables, as pease and beans. Besides the proteids, we have the fats, as in butter, milk, cheese, meats, and in certain vegetables. The fourth element consists of the mineral matters, of which but a small portion is necessary to the system. The relation of these four classes of foods, the relative proportion in which they exist in vegetable and in animal foods, their necessity for the healthful operations of the tissues of the human body, and the life energy resulting from them are not commonly known, and it would be of great benefit if more thought and attention were given to this subject. I will call your attention to this chart, which will give you a relative idea of the approximate requirements of the body of the different kinds of foods at different times in the life-history of the individual. This upper line represents the amount of carbohydrates needed; the second line the amount of proteids, and the third line the amount of fats. Now, in speaking of this, let me say that the amounts of working force may be measured in definite terms. You know that in physics you can measure the foot-pounds of any given force, and so we may measure the amount of life-energy that food will accomplish in the body. The unit of measurement in this case is denominated a calorie, and the amount of work which a calorie will do, or, in other words, the meaning of

Curves from calculations of Mrs. Ellen M. Richards, Massachusetts Institute of Technology. I. Carbohydrates. II. Proteids. III. Fats.

the word calorie is, the work required to raise the temperature of one kilogramme of water one degree.[1] This will give you something of an idea of the way we arrive at the amount of work that food will accomplish in the body. (While I am speaking I would like to pass about these charts, which have been prepared by Professor Atwater, of Wesleyan University, on this subject, showing the composition of various foods and the amount of energy which each will develop in the system.) To return to our diagram, if we take the amount of nutrients needed by individuals at different times in their life-histories we find that they vary. These vertical lines represent the age of the individual, and these horizontal lines represent the amount in grammes of the nutrients needed at the different ages. In infancy we know that starches are not needed, the stomach is not able to digest them, and that is the reason why nothing but milk is allowed to an infant. Very soon, when the teeth appear

---

[1] In one gramme of carbohydrates are 4.1 calories = 6.3 foot-pounds.
In one gramme of proteids are 4.1 calories = 6.3 foot-pounds.
In one gramme of fats are 9.3 calories = 12.2 foot-pounds.
These are tentative estimates from the researches of Professor Rubner.

and the child gets stronger, there is need for other kinds of food, and this need is best supplied by the starches, so that, according to this chart, at the age of five we find that the line of carbohydrates overtops the line of proteids by a considerable amount; that in turn overtops the fats. At ten years the proportion is not very different. But from then on the line of carbohydrates ascends rapidly and reaches its highest point at twenty-five years, and from that time it gradually falls until in old age it approaches the other lines, as in infancy. You will observe that the relative amounts ot proteids and fats needed are much less than the carbohydrates all through life.

There are many ways in which we differ in our dietaries from the people of foreign countries. It appears that more fat is eaten in this country than is eaten abroad, and we are apt to overload ourselves with too much meat. On the other hand, we are altogether given over to a too limited range in our diet. It is curious to note that we seldom find certain vegetable dishes—such, for instance, as salads other than lettuce—eaten in this country, whereas there are various other salad plants which might be eaten with advantage. Such things as cheeses are also too little used. You will sit at many a table where cheeses are taken scarcely once a week. Now, the various cheeses are very rich in proteid elements, and might profitably be added to our diet. So it is with fish. It is striking to note what a comparatively small part fish has in our diet, even in our seaport towns. In one of the tables that I passed about, Professor Atwater has given the food value, *i.e.*, the amount of "nutrients," the carbohydrates, the proteids, and the fats, also the working force, the calories, of a large number of fishes. He also shows the importance from an economic stand-point of eating more kinds of fish than we do. If you will notice the fish to be had at our markets and restaurants, you will see that it is limited to a very few kinds, such as cod, halibut, etc., whereas there are a large number of fishes in our waters that might be used.

In looking over those dietaries of different individuals, it is instructive to note that many of the hard workers, such as lumbermen, do not rely so much upon meat as one would suppose, and so it is with the German soldiers, although there is no more healthy class of men known to-day. Now, if you compare such a diet with that of our old training tables, you will very likely find a great difference in the amount of meat taken. In the training tables of years ago, and in some places nowadays, meat was the great standby, and was given at every meal almost exclusively, under the supposition that it was readily converted into muscle, that being supposed to be about all that was necessary for an athlete. Under such a course of diet we found our athletes suffering with boils and like cutaneous disturbances, largely owing, no doubt, not simply to over-exercise, but to the absolute starvation of the system for certain necessary foods. In the training tables of our college athletes of to-day, the diet does not include relatively so much meat, as various vegetables, fruits, etc., are freely eaten. It is based on a more scientific knowledge of the requirements of the system, and such matters as boils and breakdowns are practically things of the past. These dietary tables of Professor Atwater's represent the dietaries of our college athletes of to-day,—that is, our foot-ball players and oarsmen,—and are compared with dietaries of other classes of people at home and abroad.

There is one other problem of this nature which it seems wise to mention, and that is in relation to the proper methods of cooking foods. Of course, this is a very extended subject, and I will not take up much of your time with it. Our good old ranges and stoves and the like will accomplish wonders in the way of preparing our foods, but, in spite of our affection for them, they have their shortcomings. They are a particularly wise way (if the expression can be so used) of increasing the cost of cooking; in other words, our common methods of cooking are extravagant and wasteful. They are extravagant, first, in the cost of fuel, and secondly in wasting the products given off in smoke, which might be retained. A short time ago a dinner was prepared for one hundred and fifty physicians, which included various kinds of soups, escalloped oysters, various kinds of meats, vegetables, and puddings, forming an excellent dinner, and the cost of fuel—viz., oil in lamps—for cooking that entire repast was but fifteen cents. The fuel for such a dinner cooked by ordinary methods would have cost many times that amount. Much of the nutritive value of our foods is wasted for the lack of better ovens and appliances for saving the products of the substances. One method of accomplishing these results is by the use of Mr. Atkinson's "Aladdin cooker." It is rather an expensive cooker, but in time will more than save its first cost in the amount saved for fuel; moreover, cheaper forms of cookers may be used, and will answer the purpose just as well. By such a method of cooking, which is a matter of great importance, the products of cooking are particularly satisfactory. If we have well selected meat and we give it a proper length of time in our ovens, the results are usually good; but there are many persons who are unable to buy the best cuts and must be content with the inferior ones. Now, a cooker of that description, by the slow process which it necessitates,—never raising the heat above the boiling point, and steadily maintaining it,—will convert these tough steaks of our boarding-houses and cheap restaurants into food as tender as the fillet which we have been eating here. Now, that is a great thing to know. I have several times tasted meat taken from the cheaper cuts of animals, which we commonly think of as too tough to put on our tables, and have found them as agreeable to the taste as if they had been the most expensive selections. They tasted fully as good, although they did not always have the rich brown color obtained by broiling over a very hot fire: the color was somewhat paler. I do not mean to say that you should procure one of these cookers and then buy only inferior grades of meat, but where economy is aimed at, it is well to try methods that will produce methods of such great economy. This method is also very satisfactory in the cooking of the cereals and in the making of soups, and it is surprising to note the delicate flavors which are brought out. Of course, we all know that a pea soup which is cooked at home is vastly different from the partially cooked soups of our restaurants. The cause of this difference is found in the fact that in the restaurants they are cooked quickly over a hot fire, while the cooking of that kind of food should be by a slow process rather than a rapid one.

There is one other phase of the food question which I wish briefly to refer to, and, although you may be well informed in regard to it, you must remember that others are not equally so, and you might by your co-operation help us physicians very distinctly in the matter of looking out for the general health. Oftentimes patients go to you regarding their teeth who do not think themselves ill enough to need medical advice. They may perhaps have a feeling of depression or other indefinite ailments. On examining the teeth you find that they show evidence of lack of proper nutrition. It may be that they are simply run down, and a little tonic will relieve them at once, or it may be that they are practically starved for certain foods. Now, this question as to what underlies their condition is often a difficult matter, and it may be hard for you to say whether it is simply of a digestive nature or because certain substances are not being supplied, and in such cases you are doing the patients a great service if you put them on the track of being attended to; and so there are various other conditions in which the two branches, general medicine and dentistry should work together. You sometimes find that by injudicious advice various medicines have been given which tend to ruin the teeth, and it would be greatly to the advantage of the people if they could be more thoroughly enlightened as to the effect of certain mineral acids which are still prescribed by some physicians, though not so frequently as they were ten years ago, and the same might also be said of the acids associated with many foods. Certain of them may be injurious,—for instance, the acid of grapes, where the custom is to chew up the grapes. Many people also have a habit of eating lemons, which are said to be injurious to the teeth. It would be a great service to have the exact status of such facts brought clearly to their minds. And so with another allied question, the diet during the stage of lactation. Oftentimes nursing women so restrict themselves in diet as to be really starving, and if we but knew that they were ailing they might be materially benefited. Of course, it is the physician's business to look out for such matters, but in many instances he has no opportunity for doing so, as the patient does not think it necessary to seek his advice on such matters. When such cases come to you, you can do a great deal of good by your advice. It is a question of some delicacy to say exactly what attitude you should take in regard to those things. If you have studied all medical questions and have familiarized yourself with the various symptoms indicating the patient's trouble, it is your privilege to go ahead with them the same way as any one else would and prescribe medicines for them. In Massachusetts there is no prohibition in regard to this. On the other hand, in the same way that specialism exists in certain departments of dentistry, and as you refer certain work to those specially familiar with that class of work, it would be a safe rule to consider all such as special questions to be referred to a physician who is undoubtedly familiar with them, because they may not simply involve immediate effects on the teeth, but they may involve the general health of the patient; and, again, the facts in the case may be very far from what you supposed them to be. At all events, as such cases do not pertain to your own specialty you are not making any mistake by referring them to the medical practitioner.

## Figures and Tables

**Figure f1:**